# Adolescent Hormonal Contraceptive Use in the Context of Brain Development and Depression Risk: A Review and Considerations for Future Research

**DOI:** 10.1016/j.bpsgos.2026.100758

**Published:** 2026-05-15

**Authors:** Niamh MacSweeney, Taleah Dumoulin, Eira R. Aksnes, Claudia Barth, Arielle Crestol, Carina Heller, Hedvig Nordeng, Yara J. Toenders, Katharina Winkler-Crepaz, Belinda Pletzer, Adriene M. Beltz, Christian K. Tamnes

**Affiliations:** a PROMENTA Research Centre, Department of Psychology, University of Oslo, Oslo, Norway; b Division of Mental Health and Substance Abuse, Diakonhjemmet Hospital, Oslo, Norway; c Department of Psychiatry and Neurosciences, Charité Universitätsmedizin Berlin, Berlin, Germany; d Research Unit Gender in Medicine, Charité Universitätsmedizin Berlin, Berlin, Germany; e Center for Precision Psychiatry, Division of Mental Health and Addiction, Oslo University Hospital and Institute of Clinical Medicine, University of Oslo, Oslo, Norway; f Masonic Institute for the Developing Brain, University of Minnesota, Minneapolis, Minnesota; g Department of Pediatrics, University of Minnesota, Minneapolis, Minnesota; h Department of Psychological and Brain Sciences, University of California Santa Barbara, Santa Barbara, California; i Department of Psychiatry and Psychotherapy, Jena University Hospital, Jena, Germany; j PharmacoEpidemiology and Drug Safety Research Group, Department of Pharmacy, University of Oslo, Oslo, Norway; k Department of Child Health and Development, Norwegian Institute of Public Health, Oslo, Norway; l Department of Psychology, Education and Child Studies, Erasmus University Rotterdam, Rotterdam, the Netherlands; m Department of Obstetrics and Gynecology, Paracelsus Medical University, Salzburg, Austria; n Department of Psychology and Centre for Cognitive Neuroscience, University of Salzburg, Salzburg, Austria; o Department of Psychology, University of Michigan, Ann Arbor, Michigan

**Keywords:** Adolescent depression, Brain development, Hormonal contraceptives, Imaging, Women’s health, Youth mental health

## Abstract

Hormonal contraceptives (HCs) contain synthetic gonadal hormones that act on receptors widely distributed throughout the brain, thereby altering the body’s endogenous hormonal milieu in ways that may influence brain and behavior. Although HCs are among the most commonly prescribed medications for female adolescents, their effects on the developing brain and mental health remain poorly understood. This gap is concerning given that adolescence is marked by substantial hormonal change, neurodevelopment, and a sharp rise in depression risk among female youth. In this review, we synthesize current evidence on associations between adolescent HC use, depression risk, and brain structure and function. Epidemiological studies have consistently reported associations between HC use during adolescence and increased depression risk, but causal interpretation is limited by residual confounding. Neuroimaging research remains scarce, particularly in adolescents, and rarely accounts for heterogeneity in HC formulations and characteristics of use or for endogenous hormonal variation related to puberty or the menstrual cycle. We outline 3 considerations to guide future research: accounting for HC heterogeneity, incorporating developmental features of adolescent menstrual cycles, and situating HC use within its broader developmental and sociocultural context. We conclude by emphasizing the need for rigorous developmentally sensitive research to counter misinformation and better support adolescents’ reproductive and mental health care needs.

## Plain Language Summary

Hormonal contraceptives (HCs) are widely used by adolescent girls, but their potential effects on the developing brain and mental health are not well understood. This matters because puberty brings major hormonal and brain changes, and depression risk rises for girls during adolescence. Many population studies link adolescent HC use to higher depression risk, but these findings may reflect other differences between users and nonusers. In this review, we evaluate evidence linking adolescent HC use with depression risk and with brain structure and function. We highlight key research gaps and priorities to reduce misinformation about HCs and better support adolescents’ reproductive and mental health.

Hormonal contraceptives (HCs) are among the most prescribed medications for female adolescents and young adults worldwide. Beyond pregnancy prevention, they play a crucial role in managing reproductive conditions and menstrual symptoms (1). HCs contain synthetic gonadal hormones (estrogens and/or progestins) that alter systemic endogenous levels of 17β-estradiol (E2) and progesterone (P4) to prevent ovulation and/or result in local physiological changes in the uterine environment to prevent pregnancy (see Consideration 1: Heterogeneity in HCs and Mechanisms of Action for further details). As receptors for these hormones are widely distributed throughout the brain (2,3), these HC-induced changes in the hormonal milieu have the potential to influence brain and behavior.

Importantly, adolescence and emerging adulthood (ages 10–24 years) (4) represent a period of substantial neurodevelopment. Cortical gray matter volume and thickness decrease across adolescence while surface area increases during childhood and plateaus by midadolescence (5, 6, 7). Functionally, there is a shift from predominantly local connections in childhood to more distributed networks from midadolescence into adulthood (8). These changes unfold along the sensorimotor-association axis, a key organizational principle of cortical development from childhood to adolescence that reflects a shift away from mere perception and toward integration (9), which supports adolescents’ capacity to navigate increasingly complex cognitive, emotional, and social environments (10).

Beyond normative age-related changes, the adolescent brain is also shaped by hormonal changes at the onset of puberty (11). Specifically, activation of the hypothalamic-pituitary-adrenal (HPA) and hypothalamic-pituitary-gonadal (HPG) axes leads to marked increases in adrenal and gonadal hormones, including dehydroepiandrosterone, E2, P4, and testosterone (12). These endocrine shifts influence brain structure and function through adrenal and gonadal hormone receptors, modulating neuronal morphology, myelination, and synaptic organization (11,13,14). Importantly, the expression, density, and functional effects of these receptors differ between males and females, including in adolescence, and are thought to contribute to sex-specific patterns of brain development (14, 15, 16). For example, E2 and P4 receptors are highly expressed in prefrontal and limbic regions (2,3,17), brain areas implicated in emotion regulation and stress responsivity. Alterations in these emotion-related brain regions have also been associated with increased depression risk (18, 19, 20). Thus, sex differences in the timing of puberty (females typically begin 12–18 months earlier than males) and differences in hormone profiles (higher E2 and P4 but lower testosterone levels in females) (21) result in sex-specific neuroendocrine profiles (14,22,23). Consistent with this hypothesis, earlier pubertal timing, whereby this window of hormonal sensitivity is shifted earlier in development, has been associated with an increased risk for depressive symptoms in female youth (24,25). It is thought that this sex-dependent neural reorganization may underpin the 2:1 prevalence in depression that is observed in female compared with male adolescents beginning around puberty and persisting across the reproductive years (26). Together, these findings underscore how hormonal transitions during adolescence contribute to sex-specific patterns of brain development and depression risk.

Despite the widespread use of HCs in adolescence, the effects of HCs on the adolescent brain and mental health remain poorly understood, representing a knowledge gap with important clinical and societal implications. However, understanding how adolescent HC use relates to brain development and mental health requires situating it within the broader developmental and sociocultural context. Adolescents today navigate reproductive and mental health decisions in a rapidly changing society in which the online world plays a growing role. Social media platforms, especially TikTok, have expanded access to health information but have also become major sources of misinformation (27, 28, 29). Online narratives tend to exaggerate anecdotal experiences or misrepresent scientific findings about HCs, especially their potential negative effects on mood (27). While some young people experience mood-related side effects from HC use, potentially related to shared biopsychosocial risk factors, others report mood stabilization or improvement (30,31). However, this nuance is largely absent from online discourse, which can undermine confidence in HCs and deter their use among adolescents who may benefit from them. Moreover, beyond gynecological indications, HC education and access help prevent unintended adolescent pregnancy (32). Unintended adolescent pregnancy itself is associated with an increased risk of depression during pregnancy, postpartum, and later in life (33, 34, 35). Thus, left unaddressed, these knowledge gaps and misinformation could contribute to a generation of adolescents with unmet health needs, with downstream implications for reproductive autonomy and gender equality.

Although scientific interest is growing, major gaps remain in the understanding of how HCs relate to mood symptoms and brain structure and function (36,37). Epidemiological studies suggest that adolescent HC use specifically is associated with a 1.5- to 3-fold higher risk of depression diagnosis or antidepressant use (depending on HC type), but residual confounding may partially explain the observed association and limit causal inference (38, 39, 40, 41). Residual confounding refers to bias arising from unmeasured or imperfectly measured factors—such as baseline mental health vulnerability, indication for HC use, or health care–seeking behavior—through which HC users and nonusers may differ in systematic but incompletely captured ways. In contrast, neuroimaging research directly examining the association between HC use and adolescent brain structure and function is particularly scarce (36,42). This is unsurprising given the limited scope of comparable work in adult samples (43), consistent with the underrepresentation of women’s health in research (37,44). These scientific gaps are compounded by the heterogeneity of HCs themselves. Although often treated as a single category, HCs vary markedly in formulation, dose, delivery method, and mechanism of action (see Table 1). This variation has important implications for potential HC-related mood symptoms (41) and for neural influences. However, most neuroimaging studies focus on combined oral contraceptives (COCs) and do not consistently consider formulation (43). Both endogenous (e.g., pubertal development, menstrual cycle, reproductive) and exogenous (e.g., HC type and dose) hormonal milieu are therefore important to consider when interpreting associations between HC use, brain development, and mental health in adolescence.

**Table 1:** Overview of HC Types, Formulations, and Mechanisms of Action

HC Type	Hormonal Formulation	Primary Mechanism(s) of Action	Typical Delivery Type/Pattern	Ovulation Suppression	Adolescent Use Notes
Short-Acting Contraceptives					
COC	EE^a^ or newer E2/E4 + a progestin (e.g., LNG, drospirenone, desogestrel)	Negative feedback on HPG axis inhibits GnRH/LH/FSH surge; thickens cervical mucus; stabilizes endometrium	Oral; cyclic (e.g., 21 active + 7 placebo pills, which prompt withdrawal bleeding) or continuous	Consistent	Most common in HICs; also prescribed for treatment of dysmenorrhea, acne, or menstrual irregularities
Transdermal Patch, Vaginal Ring (Combined Method)	EE + a progestin (e.g., norelgestromin/etonogestrel)	Same as COCs	Weekly patch or monthly ring, cyclic (21 active + 7 pause days) or continuous use possible	Consistent	Less commonly used by adolescents
Progestin-Only Pill	Traditional forms: norethindrone 350 μg/LNG 30 μg; newer forms: desogestrel 75 μg/drospirenone 4 mg	Thickens cervical mucus; suppresses LH surge robustly in newer POPs; thins endometrial lining	Oral; mostly cyclic	Partial for traditional forms and consistent for newer forms	Most require strict adherence and must be taken at same time every day (no longer required for drospirenone-only pill); irregular bleeding commonly reported; becoming more prevalent
Injectable (Progestin Only)	Mostly DMPA 150 mg; norethisterone enanthate less commonly used	Suppresses LH/FSH; thins endometrial lining, thickens cervical mucus	Intramuscular or subcutaneous injection every 8–12 weeks	Consistent	More common in LMICs; DMPA may be associated with reduced bone mineral density in adolescence due to high E2 suppression

Long-Acting Reversible Contraceptives					
Hormonal IUD (Progestin Only)	All hormonal IUDs contain LNG but have different LNG doses (13.5–52 mg) and release rates	Primarily local: thickens cervical mucus, thins endometrial lining; small amounts of LNG enter bloodstream, which can alter HPG axis feedback in some users, especially in high-dose IUDs (e.g., 52 mg)	Intrauterine, 3–8 years depending on dose	Ovulation mostly preserved but may be suppressed initially after insertion or in high-dose IUDs	Increasing adolescent use; used to treat dysmenorrhea, heavy menstrual bleeding, and endometriosis; anxiety and pain around insertion could limit uptake
Subdermal Implant (Progestin Only)	Etonogestrel (68 mg) or LNG (2 rods × 75 = 150 mg total)	Suppresses LH/FSH (particularly in first year); thins endometrial lining, thickens cervical mucus	Up to 5 years	Mostly suppressed, but ovulation may occur in later years of use as release rates decline	Increasing adolescent use

COC, combined oral contraceptive; DMPA, depot medroxyprogesterone acetate; E2, estradiol; E4, estetrol; EE, ethinyl estradiol; FSH, follicle-stimulating hormone; GnRH, gonadotrophin-releasing hormone; HC, hormonal contraceptive; HIC, high-income country; HPG, hypothalamic-pituitary-gonadal; IUD, intrauterine device; LH, luteinizing hormone; LMIC, low- and middle-income country; LNG, levonorgestrel; POP, progestin-only pill.

a The EE prodrug estrogen mestranol was used in early COCs but is rarely used today.

The aim of this article is to guide more nuanced and developmentally grounded investigations into how HCs may modulate depression risk and neurodevelopment in adolescence. First, we review existing research on associations between adolescent HC use and depression risk and brain structure and function, drawing attention to methodological limitations. Then, we outline key considerations for future adolescent research that will inform study design.

## HCs and Depression

Across large-scale population-based studies, adolescent HC use has been consistently associated with an elevated risk of depression diagnosis or antidepressant use compared with adults and adolescent nonusers, particularly within the first 2 years of use (38, 39, 40, 41,45). Associations appear strongest for progestin-only formulations, whereas COCs generally show weaker or null associations (41). See Table 1 for descriptions of HC formulations. Recent registry studies further suggest a dose-dependent pattern among adolescent levonorgestrel-releasing intrauterine device (LNG-IUD) users, with high-dose systems (52 mg like Mirena) associated with a greater depression risk than low-dose systems (19.5 mg like Kyleena), especially when used as the first method of hormonal contraception (46,47). This finding raises important questions regarding mechanisms: One hypothesis is that higher-dose LNG-IUDs, despite their primarily local contraceptive action, result in greater systemic absorption of progestin, which could strongly influence neuroendocrine pathways. However, the precise biological mechanisms linking progestins to depression risk remain underresearched and warrant further investigation (48). Long-acting reversible contraceptives (LARCs), including the LNG-IUD, are increasingly prescribed to adolescents because of their high reliability, lack of user error, frequent use for relieving menstrual symptoms, and inclusion in some national reimbursement schemes (1,49, 50, 51, 52). Given this context, understanding potential mechanisms linking LNG-IUDs to depression risk is crucial for supporting adolescent health needs.

Although the population-level association between adolescent HC use and depression appear robust, whether adolescent HC use causes depression is far from resolved. Adolescents initiating HC use often differ from nonusers in important ways, including reasons for initiation, timing of pubertal maturation, and underlying gynecological or menstrual conditions, which may confound associations between HC use and depression (1). While some registry studies statistically adjusted for medical indications such as polycystic ovary syndrome and endometriosis (39, 40, 41), these conditions are often not diagnosed formally until late adolescence due to typical early-cycle irregularity (53,54) (see Consideration 2: Developmental Features of Adolescent Menstrual Cycles and Consideration 3: Context of Use), meaning that residual confounding likely remains. Moreover, other relevant gynecological conditions, such as premenstrual dysphoric mood disorder (PMDD) and dysmenorrhea, were not consistently considered, despite being related to mood problems (55,56). Importantly, psychosocial factors, such as stressful life events, interpersonal stress, and peer and family dynamics, are not typically captured in registry studies although these factors may influence both HC use and mental health risk (26,57).

Findings from these large-scale studies are challenged by findings from studies that have reported that COCs may have a positive effect on adolescents’ mood, especially in individuals with preexisting psychiatric conditions (58). Moreover, randomized controlled trials (RCTs) have generally reported little or no effect of HCs on mood, regardless of HC formulation (59). However, a systematic review and meta-analysis by de Wit *et al.* (59) found only 14 eligible RCTs on HCs and depressive symptoms published between 1961 and 2020 (60, 61, 62, 63, 64, 65, 66, 67, 68, 69, 70, 71, 72, 73), only one of which included an adolescent sample (69). Importantly, RCTs are prone to healthy user bias: Participants tend to be healthier than in population studies and less likely to discontinue HC use, limiting generalizability. Furthermore, adolescents who discontinue HCs due to negative side effects of HCs are especially underrepresented, further complicating efforts to disentangle adolescence as a sensitive developmental period—characterized by neurobiological and hormonal changes—from the impact of first-time HC use (74). Ethical and practical challenges compound these sampling biases further, making RCTs in adolescent populations particularly difficult to conduct.

A recent Swedish study using an emulated target trial design sought to overcome these challenges and replicated earlier findings of increased depression risk being related to adolescent HC use, particularly during the first 2 years of use (75). Notably, adolescent HC use also predicted elevated depression risk in adulthood, consistent with other findings suggesting that HC exposure during adolescence may confer lasting vulnerability to depression even after HC discontinuation (74). While these designs represent important methodological advances, they remain constrained by residual confounding, particularly psychosocial factors that influence both HC use and depression risk. Ongoing efforts to integrate causal-inference frameworks within large-scale longitudinal data will be essential for clarifying temporal and mechanistic links between adolescent HC use and depression (76).

While the increased risk of arterial thrombosis associated with HC use is well documented and routinely considered in prescribing (77), potential effects on mood symptoms remain inadequately addressed and largely absent from clinical guidelines (78,79). This omission is concerning given that mood-related side effects are the primary reason for HC discontinuation (79,80). Integrating robust evidence on HC-related mood effects into research and prescribing practices is therefore essential for aligning contraceptive care with a holistic view of adolescent well-being.

## HCs and the Adolescent Brain

Preclinical work has demonstrated that endogenous E2 and P4 exert widespread effects on the brain, influencing neuroplasticity, neurogenesis, and neurotransmission (81). E2 and P4 receptors are expressed throughout the brain, including in the amygdala, hippocampus, and prefrontal cortex (PFC) (2,3). The brain is sensitive to E2 and P4 changes over months and years, such as those occurring during puberty (11), pregnancy, and menopause (82), as well as shorter-term fluctuations across the menstrual cycle (83,84). Cyclic fluctuations have been linked to small whole-brain functional (85, 86, 87) and, to a lesser extent, structural (88) changes. Once thought to be concentrated in the medial temporal lobe (89, 90, 91), these changes are now understood to be distributed across cortical and subcortical regions, reflecting the importance of network perspectives.

Preclinical findings have also played a central role in elucidating potential mechanisms linking HCs to changes in the brain and subsequent behavior, in particular, through interactions with HPA axis functioning and stress responsivity (30,92,93). Converging with emerging evidence from humans, rodent models have shown that HCs appear to blunt cortisol/corticosterone responses to stress, which is consistent with a reduced release of glucocorticoids and altered downstream effects on stress signaling pathways (30). For example, HCs have been associated with increased circulating FK506 binding proteins (FKBP5). FKBP5 dampens glucocorticoid receptor sensitivity and modifies the negative feedback loop of the HPA axis, resulting in altered stress reactivity (94). Recent adolescent rodent models have further demonstrated HC-related changes in HPA axis function and alterations in the fold change expression of genes related to neuroimmune, hormone, GABAergic (gamma-aminobutyric acidergic), and monoamine signaling in the hypothalamus and the medial PFC (mPFC) [see (93) for details]. Although some of these HC-related changes may be specific to adolescence, such as gene expression alterations in the hypothalamus and mPFC, further research in adult rodents is required to replicate these findings. Importantly, the analogous brain regions in humans have a high density of E2 and P4 receptors and undergo substantial maturation during adolescence. Alterations in these stress- and emotion-related brain regions have also been linked to an elevated risk of depression (18, 19, 20). Together, these findings outline potential neuroendocrine mechanisms through which HCs may shape resilience and vulnerability to depression, although direct evidence in humans, especially adolescents, remains limited.

Given the well-established influence of gonadal hormones on brain structure and function (11,95) and mechanistic evidence from rodent models (88, 89, 90), the relative scarcity of neuroimaging research on HCs in humans is striking. A 2020 systematic review (96) identified only 33 structural and functional imaging studies, with just 1 including participants under 18 years (55 users vs. 55 nonusers, age range: 13.5–15.5 years) (97). Existing evidence, drawn largely from small adult samples, provides tentative and mixed findings that HC use may relate to differences in brain structure and function, although interpretations remain unclear (43,96). Methodological variability, including menstrual cycle phase, HC formulation, duration of use, small sample sizes, and small effects, likely contribute to inconsistent results (37,43). Findings on cortical thickness have been relatively consistent, with most studies reporting a localized thinner cortex, particularly within frontolimbic regions, in HC users compared with nonusers (42).

However, findings for other brain metrics have been variable. Some studies have reported lower global cortical (98,99) and intracranial volumes (100) or lower regional gray matter volumes in prefrontal [e.g., middle and superior frontal gyri (100)], temporal [e.g., anterior cingulate and fusiform gyrus (101)], and subcortical [e.g., the hippocampus (98), putamen (102), and amygdala (99)] regions. However, other studies have reported volumetric increases in some of these regions (100,101,103). Functional imaging findings have also been mixed: Some studies have reported lower prefrontal activity in HC users during emotion-processing tasks (104), while others—including the only task-based adolescent study reported to date—found higher temporal lobe activity (97). Resting-state functional connectivity findings remain inconclusive, with studies reporting both higher and lower connectivity or no differences at all between HC users and nonusers (96).

Neuroimaging research on adolescent HC use remains extremely limited. In adolescents, Marečková *et al.* (97) reported higher temporal lobe activity in COC users compared with nonusers, diverging from adult findings of reduced prefrontal activation during a similar emotion-processing task (104). More recently, Heller *et al.* conducted the first whole-brain analysis of cortical morphology in the ABCD (Adolescent Brain Cognitive Development) Study and found lower cortical thickness in the paracentral gyrus in adolescent HC users compared with nonusers after controlling for age, pubertal stage, and intracranial volume (105). Although this was the only finding surviving multiple comparison correction, the small number of HC users versus nonusers (*n* = 65 vs. 1169; mean age 14 years) reduced statistical power and generalizability. Moreover, limited data in the ABCD Study on HC formulation, duration of use, and menstrual cycle or intake phase (active or placebo pill phase) at time of scanning also restrict interpretations. Importantly, the functional significance of HC-related brain differences remains unclear, including in adults. One study in adults found that prefrontal cortical thickness differences associated with COC use were not linked to depressive symptoms (106). Considering that adolescence is a period of heightened hormonal change, brain development, and increased depression risk, this represents a critical knowledge gap.

To advance the field, a range of thoughtfully designed neuroimaging approaches is needed, collecting new data or leveraging existing datasets (18). Longitudinal and multimodal studies, whether large-scale or dense sampling, offer opportunities to examine developmental trajectories and within-person fluctuations. For example, the ABCD Study will be well positioned to examine how brain development and depression trajectories differ between HC users and a matched group of nonusers. However, it currently lacks detailed HC-related information from individuals, limiting making inferences beyond the group level. Moreover, a dense sampling study with an adult COC user demonstrated that brain structural dynamics across the menstrual cycle differed significantly from those observed in a naturally cycling individual. These differences were likely driven by the E2-dominant hormonal milieu associated with COC use (88). Extending such designs to adolescents would allow researchers to examine how endogenous and exogenous hormonal fluctuations relate to brain features and mood across the adolescent menstrual cycle and during HC use. It would also allow investigation of how these associations vary across HC types, formulations, and individuals. At the same time, well-powered cross-sectional studies can provide valuable mechanistic insights, such as by comparing adolescents using different HC formulations, delivery methods, or mechanisms of action. All new data collection should include careful characterization of HC formulation, duration and indication of use, endogenous and exogenous hormone levels, menstrual cycling, pubertal status, and psychosocial and clinical measures (37,107). Crucially, the associations between HCs, adolescent brain development, and depression must be examined within the broader developmental and social context of adolescence (see Consideration 3: Context of Use) and, where possible, using designs that strengthen causal inference, including within-person or prospective approaches and detailed consideration of potential confounders.

Existing evidence underscores that little is known about how HC use interacts with depression risk and brain development during adolescence. A lack of adolescent-specific data, methodological differences, and limited attention to the developmental and social context, all constrain interpretation of existing findings. Addressing these challenges requires a shift toward more developmentally and mechanistically grounded research frameworks. In the following section, we outline 3 key considerations to guide future research on HCs, the brain, and mental health in adolescence: 1) heterogeneity in HC formulations and mechanisms of action; 2) developmental features of adolescent menstrual cycles; and 3) the broad individual, interpersonal, and societal contexts in which adolescent HC use occurs. Together, these considerations are aimed at supporting a more nuanced understanding of how exogenous ovarian hormones may modulate adolescent brain development and depression risk and inform future study design while guiding interpretations.

## Consideration 1: Heterogeneity in HCs and Mechanisms of Action

A central challenge in interpreting findings on HCs, the brain, and depression is the substantial heterogeneity in HC active substances and formulations. As outlined in Table 1, HCs differ in hormonal composition, mechanism of action, and systemic hormone absorption, factors that may differentially influence neuroendocrine function, brain development, and mood, and accounting for this variation is essential. However, many studies, including global estimates (49,108), have not distinguished between specific HC formulations. For example, “the pill” is often reported as a single category without differentiating between COCs, which typically contain ethinyl estradiol (synthetic estrogen) and one of at least 12 progestins, whereas progestin-only pills (POPs) do not contain a synthetic estrogen. These differences are not trivial: Hormonal composition determines both the physiological mechanisms and the potential neurobiological effects of each HC type. Below, we summarize these mechanisms and refer readers to existing work for more detailed discussions (107,109, 110, 111, 112, 113).

HCs can cross the blood-brain barrier, as most synthetic forms of estrogen and progestins are sufficiently lipophilic to enter the central nervous system. Once in the brain, they bind primarily to estrogen and P4 receptors, although some progestins also interact with androgen, mineralocorticoid, and glucocorticoid receptors and can modulate GABA_A_ receptor–related neurosteroid pathways. Most HCs act through negative feedback on the HPG axis, suppressing gonadotropin-releasing hormone (GnRH) secretion from the hypothalamus. In turn, reduced GnRH lowers luteinizing hormone and follicle-stimulating hormone release from the pituitary, inhibiting follicular maturation and, for most HC types, suppressing ovulation. (Note: For progestin-only HCs, ovulation suppression can be inconsistent across formulations, and ovulation suppression is not a typical characteristic of IUDs.)

As a result, endogenous E2, P4, and testosterone levels are markedly reduced, often resembling the early follicular phase in naturally cycling individuals, although the exogenous hormones introduced by most HCs create a pharmacologically stabilized hormonal milieu distinct from natural menstrual cycling. HCs also work by thinning the endometrial lining (via mechanisms that differ somewhat between COCs and POPs), often referred to as endometrial stabilization. Moreover, HCs can thicken cervical mucus to impede sperm movement. Notably, the progestins used in HCs vary in progestational potency and androgenicity (e.g., levonorgestrel is androgenic whereas drospirenone is antiandrogenic), which can have different therapeutic purposes or psychological consequences (110,111). We also highlight that while ethinyl estradiol is the synthetic form of estrogen used in most COCs, newer formulations can contain estradiol hemihydrate (bioidentical to endogenous E2) or the estradiol ester prodrug estradiol valerate. More recently, estetrol, a naturally occurring estrogen produced by the human fetal liver during pregnancy, and proposed to have fewer cardiovascular risks, is used (114).

## Consideration 2: Developmental Features of Adolescent Menstrual Cycles

Understanding menstrual patterns typical of adolescence is essential for interpreting the effects of HCs on adolescent brain development and depression risk. Exogenous hormones introduced via HCs, and the accompanying suppression of endogenous hormones depending on formulation (see Table 1), may interact with or obscure the typical hormonal fluctuations that occur as neuroendocrine systems change. Such interactions could complicate interpretation of neurodevelopmental and mood effects related to HC use.

Adolescence is characterized by the ongoing maturation of the HPA and HPG axes, which regulate the increasing production and activity of adrenal and gonadal hormones, respectively (12,115). Within these broader neuroendocrine changes, menarche (first menstrual period) is a salient milestone in female development. It typically occurs between ages 12 and 13 (116,117), although age at menarche has been declining across recent decades (117,118). The years following menarche are marked by substantial hormonal variability, including irregular cycles, as feedback mechanisms within the HPG axis continue to mature (119). Early cycles are often anovulatory—up to 85% within the first 2 years postmenarche—resulting in low P4 levels and wide variability in cycle length (111,120, 121, 122). A systematic review of adolescent menstrual patterns (120) reported mean cycle lengths of around 34 days, with most cycles ranging from 21 to 45 days. Menstrual cycles tend to shorten and become more regular across adolescence, but it can take up to 6 years postmenarche to stabilize, with adult norms of 21 to 35 days (123,124).

Although individuals vary in the length and pattern of their menstrual cycles, a mature cycle typically involves distinct phases: 1) follicular, marked by steadily rising E2 as ovarian follicles mature, peaking just before ovulation; 2) ovulation, triggered by LH, the dominant follicle releases an egg; and 3) luteal, when the corpus luteum (remnant of the dominant follicle) secretes E2 and P4. In the absence of fertilization, the corpus luteum regresses causing E2 and P4 to fall rapidly, resulting in menses, marking the start of a new cycle. These recurring fluctuations create a dynamic environment that remains in flux as neuroendocrine feedback mechanisms undergo refinement during adolescence (12).

This developmental and individual variability has direct implications for research on HCs, the adolescent brain, and depression (125). For example, the degree of endogenous hormone suppression, varying across HC formulations, may influence how exogenous hormones interact with the refinement of the HPG axis during adolescence, which could in turn influence structural and functional brain development. Moreover, adolescence is a period of heightened hormone sensitivity, and thus neurodevelopmental and mood changes may be similarly related to hormone variability rather than to absolute concentrations (126). Furthermore, individuals may differ considerably in their degree of hormonal sensitivity due to a combination of biological and environmental factors. As discussed in Consideration 3: Context of Use, it is therefore important to contextualize how HC use relates to brain development and depression risk.

Although variability in adolescent menstrual cycles can pose challenges for research (121), menstrual cyclicity offers a valuable, noninvasive window into both reproductive and general health. The menstrual cycle is increasingly recognized as a vital sign—an indicator of underlying physiological and psychosocial well-being (123). Features such as cycle length, pain, bleeding patterns, hypermenorrhea, or mood changes can reflect individual differences in hormonal regulation and could point to early signs of gynecological problems as well as to hormonally driven mood disorders such as PMDD. Intensive longitudinal designs integrating daily hormone assays (see Box 1), menstrual cycle characteristics, and mood tracking are needed to ascertain how menstrual cycle regularity stabilizes during adolescence and how variability may signal risk for gynecological or mental health conditions. This is particularly important in the study of HCs, as they are often prescribed to treat these conditions (1). This work in turn could inform more personalized contraceptive counseling that integrates individual, developmental, and mental health risk profiles, and importantly, maintains follow-up to monitor the emergence and trajectory of mood symptoms after initiating or switching HCs.

Box 1 **Measuring Hormones and Menstrual Cycle Dynamics**

**Table:** 

Measuring endogenous hormonal fluctuations (i.e., E2, P4, LH, FSH) across the menstrual cycle and exogenous hormones introduced by HCs is crucial for linking endocrine dynamics to adolescent neurodevelopment. However, heterogeneity in biospecimens, assay types, and sampling frequency can limit interpretability (82). Below, we outline considerations to improve reliability in developmental neuroendocrine research. Biospecimens • Blood-based measures remain the gold standard but are invasive and often impractical for repeated sampling (149). • Saliva and urine are less invasive alternatives, although hormone concentrations are typically lower. Urine measures are also affected by dilution and therefore require normalization to creatinine (149,150). • DUS methods allow feasible daily sampling with precision comparable to blood or liquid urine (121,150,151). Cycle staging and sampling • Peripubertal cycles can often show irregular ovulation, variable lengths, and large fluctuations in hormone concentrations. • When vaginal ultrasound is not feasible, ovulation can be inferred by an LH peak followed closely by a rise in progesterone (<7 days) (121). • Daily collection at consistent times (up to approximately 45 days) can help detect individual-specific hormonal surges and account for cycle-length variability (82,111,121). • Less intensive designs, such as detecting LH peaks within each cycle, can be more practical but provide less cycle staging (121). Assay considerations • Immunoassays are cost efficient and user friendly but may show cross-reactivity and reduced specificity (152,153), particularly for exogenous hormones as these molecules can differ structurally from their endogenous counterparts and may not be detected by assay antibodies (111,154). • MS offers greater specificity and sensitivity across biospecimens (including DUS) (155), enabling simultaneous detection of multiple hormones from lower sample volumes and circulating concentrations (150,151). Such precision is particularly well suited for studies evaluating menstrual cycle dynamics and comparing hormonal profiles between naturally cycling and HC users (154). • Methodological advancements continue to improve MS sensitivity and accessibility, facilitating more precise hormone assessments across developmental and contraceptive contexts.

DUS, dried urine spot; E2, 17β-estradiol; FSH, follicle-stimulating hormone; HC, hormonal contraceptive; LH, luteinizing hormone; MS, mass spectrometry; P4, progesterone.

## Consideration 3: Context of Use

**Figure 1:**
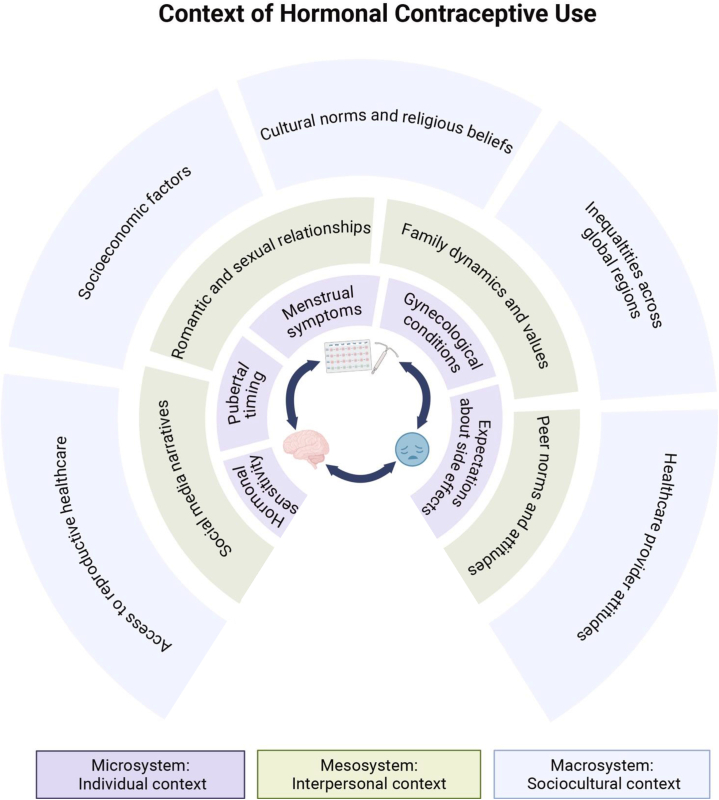
The confluence of hormonal contraceptive use, brain development, and depression risk in adolescent females occurs within a developmental ecosystem of individual, interpersonal, and sociocultural factors. (Figure created in BioRender.)

Adolescent HC use does not occur in isolation. Inspired by Bronfenbrenners’s Ecological Systems Theory (127) and as illustrated in Figure 1, we conceptualize HC use as embedded within interacting individual (micro), interpersonal (meso), and sociocultural (macro) systems that jointly shape who initiates HCs, which methods are chosen, and how use and side effects are experienced. We selectively highlight contextual factors most relevant to adolescent depression and brain development, noting that this is not a comprehensive review.

### Microsystem: Individual Factors

Gynecological conditions are common in adolescence but are often underdiagnosed and underresearched, despite their substantial impact on emotional, social, and academic functioning (55,128, 129, 130). For example, primary dysmenorrhea, the leading cause of school absenteeism among adolescent females (55,128,131,132) and heavy menstrual bleeding, more frequent in adolescence than adulthood (133), are associated with reduced participation in social activities and sports and higher depressive symptoms (128,134). However, these symptoms are often normalized as part of growing up and managed without medical consultation (130). Given the significant physical and emotional changes associated with puberty, menstrual symptoms can amplify vulnerability to distress and negatively impact well-being (135). The scarcity of adolescent-specific reproductive health research limits our ability to characterize the full spectrum of menstrual cycle patterns in adolescence, including symptoms that affect functioning and well-being regardless of whether they signal underlying pathology (136). In turn, this constrains research on brain development and mental health, as unrecognized hormonal variability may obscure key associations (82,107) and may limit how well clinical guidance reflects adolescent-specific developmental physiology (123). At the same time, HCs are often prescribed to adolescents for noncontraceptive indications, such as managing heavy menstrual bleeding, dysmenorrhea, and endometriosis (1,137). These therapeutic uses play a central role in adolescent health care, improving quality of life and daily functioning. As such, it is important to recognize both the clinical advantages of HC use and the need to balance them with careful evaluation of potential neuroendocrine or mood-related effects. Understanding how endogenous hormonal changes, gynecological conditions, and the exogenous hormones introduced by HCs interact during this sensitive developmental period is therefore critical for interpreting the broader effects of HC use on adolescent brain and mental health.

Beyond gynecological factors, individual differences in genetic propensity for mental health conditions, pubertal timing, menstrual cycle regularity, hormonal sensitivity, and neurodevelopmental conditions may influence, and be influenced by, initiation and experience of HC use. For example, individuals who initiated HCs before the age of 19 had a higher polygenic score for depression and attention-deficit/hyperactivity disorder (ADHD) on average (138). These findings suggest potential genetic confounding. However, the interplay of genetic factors with HC use, depression risk, and brain development remains largely unexplored and represents an important direction for future research. Moreover, oral contraceptive use among female adolescents with ADHD may compound an already heightened risk for depression in this group (139). Knowledge and beliefs about HCs can also affect uptake and adherence (57); concerns about mood-related side effects, potentially amplified by online misinformation (27, 28, 29), may shape expectations, bias self-reported outcomes in research studies, and confound findings. Future studies should also assess antidepressant and other psychotropic medication use, as antidepressant prescriptions are often used as a proxy for depression diagnosis in epidemiological studies. However, the pharmacological and behavioral interactions between HCs and antidepressants remain poorly understood and should be investigated as they could potentially aid the development of tailored treatments. Together, these individual-level influences are important to consider in studies linking HCs to neurobiological or mental health outcomes.

### Mesosystem: Interpersonal Context

Decisions around HC use are embedded within interpersonal environments, and shaped by partners, family, peers, and increasingly, online influencers. Partner influence is strong, reflecting entrenched gender norms that position contraception as women’s responsibility, subject to male approval (57,140). Family attitudes, particularly parental disapproval of adolescent sexual activity, can deter HC use or encourage secretive HC use (57). Many adolescents may also feel embarrassed or reluctant to discuss contraception with their parents and therefore rely on peers or online sources for information.

While peers, family, and online spaces can promote autonomy and shared learning among adolescents, anecdotal experiences from these sources often carry more weight than medical advice (27,57,141). Social media, in particular, can empower youth with accessible reproductive health information, but it can also amplify misconceptions surrounding HCs, including overgeneralized and exaggerated claims about physical side effects, infertility, and cancer risks (27, 28, 29,142,143). Furthermore, social media has become a prominent space for self-diagnosis and discussion of mental health conditions (144), highlighting broader shifts in how young people think about and engage with their psychological well-being. These evolving narratives extend to how young people interpret and report their mental health experiences in relation to HC use, which should be considered by researchers (18). Together, interpersonal and online influences can both facilitate and constrain HC use, shaping the socioemotional environment in which HC initiation and (dis)continuation occur.

### Macrosystem: Sociocultural Context

Patterns of HC use vary widely across geographic regions, reflecting differences in reproductive health care access and cost, education, knowledge about reproductive rights, and sociocultural norms (57). HC use is highest among adolescents in high-income countries (HICs) and lowest in low- and middle-income countries (LMICs) (49). Furthermore, use of HC types also varies regionally: OCs remain the most used HC by adolescents in HICs, although the use of LARCs has increased recently (50,51,145). In LMICs, OCs, injectables, and implants are the most prevalent HCs used by adolescents (146). Importantly, adolescent-specific epidemiological data remain limited globally, constraining granular comparisons by formulation or sociodemographic factors. For example, reports from the United Nations Department of Economic and Social Affairs typically aggregate contraceptive use across broad age bands (e.g., 15–19 and 20–24 years) and method categories (e.g., modern vs. traditional), without age-specific breakdowns by contraceptive type (49). Nonetheless, this regional variability underscores the importance of considering the sociocultural context when studying HC use. For example, judgmental attitudes and lack of specialized knowledge on HCs from health care providers can deter use (147). Broader societal structures and norms regarding, for example, religion, premarital sex, and gender roles also shape whether and how adolescents initiate, continue, or discontinue HC use.

Importantly, societal stigma and systemic barriers not only limit HC access but also contribute to unmet health needs for adolescents with gynecological conditions or menstrual problems for which HCs are commonly prescribed (1,148). These macrolevel influences intersect with individual and interpersonal contexts to shape who uses HCs, for what reasons, and how their effects—both physiological and psychological—are experienced and reported. Recognizing these broader determinants is critical for situating research on adolescent HC use, brain development, and mental health within a global and developmental framework.

## Conclusions

This is a pivotal moment in women’s reproductive health research, especially for adolescents and young adults. Advances in developmental neuroscience, including large-scale longitudinal studies and investigator-led deep phenotyping studies, provide unprecedented opportunities to conduct rigorous, transparent, and developmentally sensitive research that can transform the understanding of how HCs interact with the adolescent brain and mental health. Such progress is urgently needed, not only to improve scientific understanding but also to stem the rising tide of misinformation and empower young people to make their own informed decisions about their reproductive and mental health.
